# “Just trying to make it work:” A qualitative exploration of healthcare provider implementation considerations for integrating mental health and HIV care in central Uganda

**DOI:** 10.1371/journal.pone.0353859

**Published:** 2026-08-14

**Authors:** Divya Gumudavelly, John Baptist Kiggundu, Florence Ayebare, Christine Kiwala, Joel Senfuma, Anne Katahoire, Carol Birungi, Jeremy Schwartz, Isaac Ssinabulya, Noeline Nakasujja, Chris T. Longenecker, Ashley Hagaman, Fred C. Semitala, PULESA-Uganda study

**Affiliations:** 1 Yale School of Public Health, Department of Social and Behavioral Sciences, New Haven, Connecticut, United States of America; 2 Uganda Initiative for Integrated Management of Noncommunicable Diseases, Kampala, Uganda; 3 Infectious Diseases Research Collaboration, Kampala, Uganda; 4 Infectious Diseases Research Collaboration, Kampala, Uganda; 5 Infectious Diseases Research Collaboration, Kampala, Uganda; 6 Infectious Diseases Research Collaboration, Kampala, Uganda; 7 Makerere University, College of Health Sciences, Kampala, Uganda; 8 Makerere University, Joint AIDS Program, Kampala, Uganda; 9 Yale School of Medicine, Section of General Internal Medicine, New Haven, Connecticut, United States of America; 10 Uganda Initiative for Integrated Management of Noncommunicable Diseases, Kampala, Uganda; 11 Uganda Initiative for Integrated Management of Noncommunicable Diseases, Kampala, Uganda; 12 Makerere University, College of Health Sciences, Department of Psychiatry, Kampala, Uganda; 13 University of Washington, Division of Cardiology and Department of Global Health, Seattle, Washington, United States of America; 14 Yale School of Public Health, Department of Social and Behavioral Sciences, New Haven, Connecticut, United States of America; 15 Makerere University, College of Health Sciences, Department of Internal Medicine, Infectious Diseases Research Collaboration, Kampala, Uganda; University of the Witwatersrand, SOUTH AFRICA

## Abstract

**Background:**

There is a growing push to integrate mental health services within HIV care in Uganda. Given the limited mental healthcare infrastructure in the country and growing burden of mental health issues among the HIV positive patients, there is a critical need to understand 1) What are the current practices, routines, barriers, and facilitators that influence providers’ ability to address their patients’ mental health needs? 2) How do providers navigate this environment to provide mental healthcare?

**Methods:**

Semi-structured qualitative interviews were conducted with 16 healthcare providers at two HIV clinics to understand current practices, routines, barriers, and facilitators to implementing mental health care services within HIV care. Abductive thematic analysis, drawing from Consolidated Framework for Implementation Research (CFIR) and Theoretical Domains Framework (TDF) frameworks, was used in conjunction with inductive analytical approaches to explore this research question. Both frameworks guided the development of the interview guide and the creation of the codebook. The CFIR was used to elucidate and understand systemic and institutional barriers and facilitators to mental health service integration and the TDF was used to determine provider level processes that impact the uptake of mental health protocols in practice.

**Findings:**

In lieu of formalized integrated mental health services, HIV care providers navigate a complicated implementation climate and experience tension with both the clinic they work for and the patients they serve. They overcome these complications with a range of adaptive processes, collectively summarized as “making it work.”

**Conclusions:**

These findings highlight the hidden mechanisms HIV care providers engage in to cater for patients’ mental health needs and demonstrate the impact of this work on their own wellbeing as well.

## Introduction

The HIV/AIDS epidemic is a major public health concern, presently impacting more than 39 million people globally [1]. Nearly 70% of those impacted by HIV/AIDS reside in southern and eastern Africa [2]. Uganda has demonstrated significant strides in reversing, and ultimately mitigating, its HIV epidemic. This improvement is depicted in the drastic decline in the prevalence of HIV from 30% in the early 1990s to 5.8% presently [3]. These achievements can be attributed to the highly decentralized nature of HIV care in clinic and community based settings, robust health education efforts, external vertical funding from sources such as the US President’s Emergency Plan for AIDS Relief in Africa (PEPFAR), and internal policy prioritization [4].

Because of these successes, people living with HIV (PLHIV) are aging and living longer [5]. They are subsequently at risk for developing other chronic comorbidities, including mental health and other noncommunicable disease (NCD) conditions. The interaction of the HIV and NCD epidemics pose significant negative risks to patients. For example, in Uganda, the prevalence of mental health conditions like depression is nearly 10 times higher among PLHIV than the estimated prevalence in the general population [6]. Additionally, in this region, there is a high prevalence of hypertension (HTN) among those PLHIV who are heavily treated on antiretroviral therapy (ART) [7]. These mental health and hypertension comorbidities complicate the ability of PLHIV to adhere to medications, and subsequently, impact the ability to which providers can help their patients achieve viral suppression. Prior research reveals that in lower-resourced contexts, treating patients with multiple comorbidities without the necessary infrastructural support leads to providers offering siloed care, or handling multiple clinical priorities and experiencing burnout [8]. Consequently, there has been a push to transform HIV clinics into comprehensive healthcare hubs and a wealth of evidence suggests that integrating NCD care within vertical HIV programming is feasible [9]. Several implementation studies reveal that screening for NCDs like hypertension can destigmatize these conditions among the general population and concentrate necessary resources in a single, public based setting [9].

Despite the high prevalence of depression, alcohol use disorder, and other mental health conditions among PLHIV and recommendations put forth by the Uganda National HIV and AIDS Strategic Plan for providers to address this burden, efforts to integrate mental health services within HIV care in Uganda are not as widespread [10]. This can ultimately complicate long term care of PLHIV, as the mental health infrastructure in Uganda is sparse and unable to meet the vast, unique mental health needs of PLHIV [11–13]. Other formative qualitative literature suggests that providers perceive mental healthcare as intertwined with HIV care and necessary for the improvement of HIV related outcomes [14]. Similarly, an additional study gauging stakeholder perspectives in Uganda has identified that in spite of added workload, delivering integrated mental health and HIV services improved the providers’ ability to cater to holistic patient needs [15]. Previous studies in Uganda have shown that HIV care providers prefer protocolized depression treatment algorithms more than relying on clinical acumen to refer PLHIV to adequate mental health services [15]. Additionally, other efforts to integrate depression care for adolescents within clinic and community models of HIV care found that parental support eased the burden on providers to offer care [16]. This suggests that effective mental health care delivery strategies depend on organized clinical efforts as well as leveraging external factors like family support as well.

Given this context and the policy to practice gap between the government putting forth recommendations and having them sustainably implemented in practice, there is a critical need to understand the organizational cultures that can promote the longevity of such service implementation for mental health issues broadly. Specifically, it is important to understand the shared values, practices, routines, and resources within the clinical setting that can support or hinder mental health service integration efforts. Understanding this implementation climate and how providers operate within it can provide better insight for how new protocols can be developed and implemented. Moreover, given the highly decentralized and integrated nature of HIV care with other NCDs, like hypertension, there is a critical need to understand these processes within the context of already integrated care efforts. This perspective allows for a more realistic and grounded understanding of how mental health services can be introduced in this climate and implemented in real-world clinical practice.

## Current Study

The current study aims to address this policy to practice gap through understanding HIV care providers’ experiences within the context of integrated HTN care. Given that the providers had very recent experiences of integrating hypertension with HIV care, their insights into mental health integration were often framed within their experiences delivering integrated hypertension and HIV care. While hypertension is not a focus of the analysis, it serves as an important implementation context through which mental health services can be delivered. With this context, the study explores the following questions: 1) What are the current practices, routines, barriers, and facilitators that influence providers’ ability to address their patients’ mental health needs? 2) How do providers navigate this environment to provide mental healthcare?

## Methods

### Study setting

This qualitative study exploring mental health service integration was embedded and conducted within Strengthening the Blood Pressure Care and Treatment cascade for Ugandans living with HIV-ImpLEmentation Strategies to Save lives (PULESA-Uganda), an NIH National Heart, Lung, and Blood Institute funded study evaluating the integration of HTN care within existing HIV services in HIV clinics in Uganda (NCT05609513). This mental health qualitative study was conducted during the formative phase of PULESA-Uganda, wherein 2 pilot sites were implementing integrated HIV/HTN packages. Clinic A is located within a public sector health facility in Wakiso district which also offers outpatient care, maternal and child health services, vaccination with separate HIV and HTN clinics. The facility is headed by a medical clinical officer (similar to advanced practice providers in the U.S.) and the staff include nurses, medical clinical officers, laboratory technicians, data officers, pharmacy technicians, and HIV peer counselors. Clinic B is located within a private not for profit (PNFP) hospital in Wakiso district. Although the hospital has specialist and non-specialist physicians, the HIV clinic team comprises medical clinical officers, nurses, laboratory technician and HIV peer counselors. HIV services at both clinics are supported by local PEPFAR implementing partners. HTN services were integrated into HIV care at both clinics during the PULESA-Uganda pilot phase. This meant that PLHIV were screened for hypertension, diagnosed, prescribed, and dispensed medication free of charge, and assessed for adherence and hypertension-related complications by the same healthcare providers and at the same points where they received HIV-related care.

### Data collection procedures

We purposively sampled and recruited healthcare providers across both study sites to participate in a single elicitation qualitative interview exploring their perceptions on the feasibility of integrating mental health services within HIV care. Participants were eligible if they 1) had responsibilities in delivering services for HTN services for PLHIV and 2) had responsibilities in providing mental health services. Providers were excluded based on having professional responsibilities mostly related to storing and documenting patient data. Given the dynamic nature of HIV service delivery, where providers must step outside their designated roles to support understaffed areas of the clinic, we did not sample by job title.

The interview guide was developed by co-authors DG, AH, JIS, CB, and AK. The final guide included the following key domains: provider background and responsibilities, conceptualization and orientation to mental health, confidence in appraising mental health problems, and perceptions on organizational capacity for mental health service integration. Some of the interview questions were deductively derived from the *Inner Setting* and *Outer Setting* domains outlined in the Consolidated Framework for Implementation Research (CFIR) [17] and the *Knowledge*, *Skills, Social/Professional Role and Identity*, and *Motivation and Goals* domains from the Theoretical Domains Framework (TDF) [18]. Others were inductively constructed based on observations of clinics and insights from co-authors JBK, CK, JIS, FCS, and CL.

Data collection occurred between July 7 and August 3, 2022. The lead author (DG) conducted the interviews in English. All interviews were conducted at the providers’ place of work. DG audio taped the interviews and kept field notes at the end of each interview, tracking her positionality and summarizing key points from the participants’ narratives. Interviews were subsequently debriefed with the co-authors FA, JBK, CK, JIS, and FCS. These conversations helped explore the reliability and validity of the findings and helped contextualize findings within mental healthcare and HIV care in Uganda. On average, the interviews were 45 minutes in length, ranging from 30 to 60 minutes overall. The final interview guide can be found in S1 File.

### Data analysis

**Fig 1:**
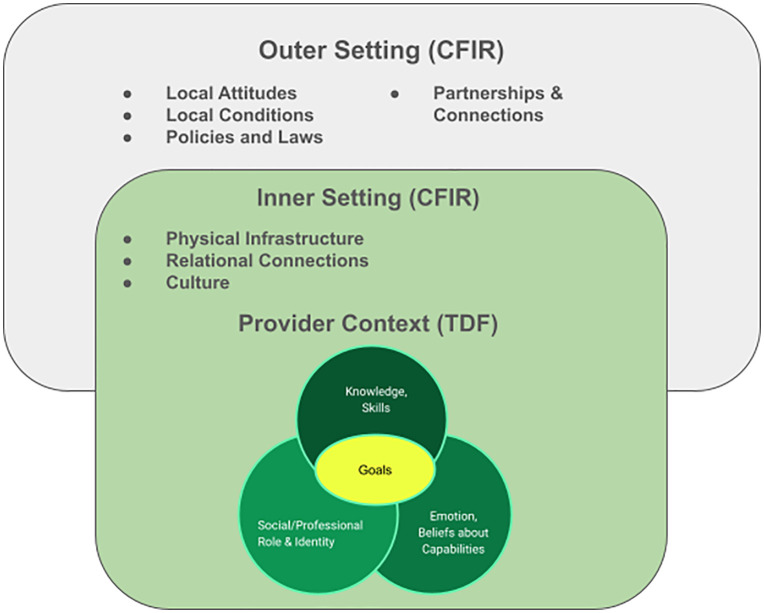
Relationship among CFIR and TDF Constructs Used in Analysis.

Concurrent with and after data collection, DG transcribed and open coded the interviews. An initial iteration of the codebook was generated through research team discussions, with a combination of inductive codes emerging from the interviews and deductive codes rooted in the CFIR and TDF constructs. For example, inductively, we coded for cultural perceptions of mental health among the provider population and challenges providers encounter when interacting with patients’ family members. Deductively, we coded for inner and outer setting features of the HIV clinics as well as provider level knowledge and confidence around appraising mental distress. Co-authors DG and FA independently piloted the codebook with 6 interviews and discussed coding discrepancies and ideas that were not being captured. A discrepancy that emerged at this stage of the analysis was whether to code provider perceptions of mental health differently from providers’ understandings of mental health protocols within the clinic. The authorship team decided to code provider perceptions separately from provider understanding to identify nuances between individual understandings of mental healthcare and organizational capacity for delivering related services. Fig 1 below demonstrates which CFIR and TDF constructs were used in the analysis, and how they relate to one another to paint a picture of the larger implementation climate.

After the coding agreement was achieved, DG independently coded the dataset using NVivo 20. The authorship team and field notes were regularly consulted to establish context. For example, CK provided insights on how patients assume somatic manifestations of anxiety and depression as similar to other physical health conditions. DG kept annotations and memos throughout the coding process to track analytical insights and emerging themes. Theme development centered around how providers adapt within resource constraints to address complex patient needs and fulfill clinical obligations.

Iterative coding and data debriefs concurrent with data collection led the research team to believe that data saturation was achieved. These procedures allowed the team to identify additional care delivery perspectives that were missing, like those of a laboratory technician, as well as implementation topics, like differentiated service delivery mechanisms.

While DG was the primary analyst formally coding the interviews, several strategies were employed to ensure analytic rigor and trustworthiness throughout the study. As mentioned, transcripts and initial open codes were collaboratively reviewed by members of the research team to iteratively refine the codebook. This ensured that DG applied each code consistently throughout the dataset, while maintaining analytic continuity as the primary coder. Data and coding debriefs with those who developed the codebook helped verify this as well. Given the interpretive nature of qualitative inquiry, the authorship team did not prioritize quantitative measures like Cohen’s kappa or interrater reliability. Contemporary qualitative methodologists have noted that such quantitative metrics do not always reflect interpretive approaches to thematic data, and that thematic analysis extends beyond multiple coders simply replicating a coding process. The authorship team instead emphasized the level of engagement with the data and transparency of analytic decision making throughout the coding process [19].

### Statement of reflexivity

The study team consisted of Ugandan and American researchers, with varied backgrounds in public health, clinical medicine, and medical anthropology. Notably, the lead author and primary interviewer for this study is an Indian American who comes from a different cultural background from the study participants and context. This outsider perspective was balanced through extensive rapport building with the study team and participants, which helped challenge any underlying assumptions.

### Statement of ethics

Written consent was obtained from all participants prior to their involvement in the study. All elements of the study, including the interview guide, methods, and participant consenting process, were ethically approved by the Institutional Review Boards at Yale University (New Haven, Connecticut, USA) (IRB Protocol ID: 2000032922) and Makerere University School of Medicine Research and Ethics Committee (Mak SOMREC) (Kampala, Uganda) (IRB Protocol ID: REF Mak-SOMREC-2021–58). The study also received clearance from Uganda National Council of Science and Technology (UNCST). All paper consent forms were kept in lockable cabinets only accessible to the study team and all audio-visual elements were stored in a data-encrypted, HIPAA compliant cloud storage system.

## Results

Sixteen (6 at Clinic A, the public sector facility and 10 at Clinic B, the PNFP facility) interviews were conducted across both study sites. Most of the participants (n = 13, 81%) were female and 56% were nurse counselors. A detailed description of the study sample can be found in Table 1.

**Table 1:** Participant demographics.

PID	Clinic	Sex	Age (years)	Job Title
1	A	Female	20 - 30	Psychosocial Counselor
2	A	Female	20 - 30	Nurse Counselor
3	A	Female	20 - 30	Nurse Counselor
4	A	Male	40 - 50	Psychiatric Clinical Officer
5	A	Female	31 - 40	Nurse Counselor
6	B	Female	20 - 30	Nurse Counselor
7	B	Female	40 - 50	Clinical Officer
8	A	Male	20 - 30	Clinical Officer
9	B	Male	20 - 30	Clinical Officer
10	B	Female	20 - 30	Nurse Counselor
11	B	Female	41 - 50	Psychosocial Counselor
12	B	Female	41 - 50	Nurse Counselor
13	B	Female	20 - 30	Nurse Counselor
14	B	Female	31 - 40	Laboratory Technician
15	B	Female	31 - 40	Nurse Counselor
16	B	Female	31 - 40	Nurse Counselor

### “Making it Work:” The invisible labor that overcomes institutional, staff, and patient challenges

It is important to note that participants frequently referenced hypertension integration efforts when discussing potential efforts to integrate mental health care, given their prior and current experiences with these efforts and the similar infrastructural demands required. Although hypertension is not a focus of the study, references to it will appear throughout the interviews as a lens through which the providers interpret mental health service integration. All the providers mentioned specific barriers and facilitators to delivering mental healthcare at the institutional level, the staff level, and at the PLHIV level. Mental health-specific clinical protocols were largely absent, and providers reported differential levels of exposure and training to mental health services. While some nurses received formal psychosocial training in their education, others recall never receiving formal or practical training. However, several resources were embedded in the HIV care system that providers drew upon to support patients with mental distress. These included peer counselors (also referred to as expert clients) who provided psychosocial support and follow-up services, as well as nurse counselors trained in adherence counseling, which often served as a mental health intervention. One clinic also had a part-time psychiatric clinical officer available on select days. This resource context greatly informed the providers’ ability to provide mental health care. Collectively, these barriers and facilitators paint a larger picture of the implementation climate for mental healthcare service integration within HIV clinics. They are highlighted in Table 2.

**Table 2:** Institutional, staff, and patient level barriers and facilitators to MH service provision within HIV care.

Level	Barriers	Facilitators
**Institutional** (HIV Clinic) CFIR Constructs: Inner Setting, Implementation Climate	Inadequate supply of mental health medications	Embedded psychosocial/adherence counseling
	Insufficient mental health referral systems	Peer clients/counselors providing additional support
	Insufficient private spaces, protocols, and comprehensive documentation platforms for mental health	Weekly ‘audit meetings’ to discuss viral suppression among patient populations
**Staff** (Nurse counselors, etc.) TDF Constructs: Knowledge/Skills, Social/Professional Role, Emotion/Beliefs, Goals/Motivation	Training needs include identifying and addressing mental health problems	Counseling as a fundamental professional responsibility to the patient
	Significant workload	Empathy as a personal responsibility to the patient
	Difficulty establishing trust and relatability with patients	Wide ranging schematic perceptions of mental illness
		Religious and innovative problem-solving strategies to supplement adherence counseling
**Patient** (PLHIV) CFIR: Outer Setting, Implementation Climate	Poverty/low socioeconomic status	Supportive family structure
	Discrimination in social settings	Successful adherence to HIV medication
	Community perceptions of mental illness as ‘*omulalu’* and how it can be induced through bewitchment	

The providers identified these barriers and facilitators based on their experiences in adopting and integrating HTN care within HIV care. They perceived many of the barriers and facilitators to successfully integrating HTN as the same to successfully integrating mental health within HIV care. For example, supportive family structure is a patient-level facilitator that eases their ability to improve mental health and HTN health outcomes for PLHIV. Likewise, high workloads are a provider-level barrier to delivering integrated HTN/HIV and mental health/HIV care long term.

**Fig 2:**
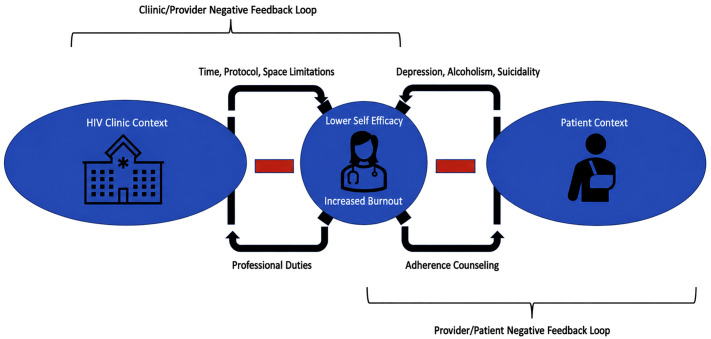
The Impact of “Making it Work” on HIV Healthcare Providers in Central Uganda.

An important deviation, however, is the clinics’ logistical set up and documentation systems. The providers noted that the existing clinical infrastructure is sufficient to adopt HTN services, as they “*can openly measure the patient’s blood pressure” (PID 9, Clinic B, Clinical Officer)* without relying on private spaces, since HTN is less stigmatizing than mental health. For the purposes of mental health service integration, however, this clinical infrastructure is a barrier. They reported that mental health services require *“more privacy to ask sensitive questions” (PID 7, Clinic B, Clinical Officer)* which the clinics do not presently have. Our analysis reveals that the healthcare providers, in negotiating the HIV clinics’ context and patients’ context described in Table 2 to deliver mental healthcare services, are at the center of two negative feedback loops. These relationships are summarized in Fig 2.

The first negative feedback loop is between the providers and the HIV clinics. The HIV clinics’ implementation features (time, protocol, and space limitations) shape the limited resources available to the provider to deliver mental healthcare services. The providers experience tension when they operate within these constraints to meet performance expectations and obligations to the HIV clinic. The second negative feedback loop is between the providers and their patient population. PLHIV present to the provider a wide range of psychosocial problems, from overt behaviors like excessive talking and alcohol dependence to more invisible mental health problems like depression and suicidal ideation. Providers negotiate these problems through adherence counseling, which is often limited to a select few PLHIV with poor adherence and is insufficient in detecting, addressing, and treating them. A clinic officer at Clinic B explains the cumulative impact of navigating these negative feedback loops:

*“But it’s not your fault sometimes. You have to counsel yourselves that ‘it’s not my fault’. You have to share it with your team that you are trying your best, you are just trying to* ***make it work****.” (PID 7, Clinic B, Clinical Officer)*

The remaining results will elucidate how the features highlighted in Table 2 interact with one another to create these negative feedback loops. It will also explore the process of how providers leverage clinic, staff, and patient level facilitators to “make it work,” highlighting the invisible labor they engage in to overcome the barriers highlighted in Table 2. TDF was used to make meaning of the provider and their own context, namely how their confidence and self-efficacy is impacted as they navigate these negative feedback loops. The CFIR was used to elucidate the inner setting (e.g., clinic infrastructure, protocols, and documentation systems), the outer setting (e.g., community stigma, resource availability, patient socioeconomic context), and implementation climate (e.g., readiness for change). This framework allowed us to situate provider narratives within broader contexts of the HIV clinics and patients’ lived realities.This deductive analysis was complemented with inductive analysis to understand providers’ experiences in “making it work:” the unrealistic demands placed upon both the providers and their patients and how these take a mental toll on the provider as well.

### Providers strain professional boundaries to ensure PLHIV achieve viral suppression

Providers frequently contend with barriers on the HIV clinic level (insufficient private spaces, protocols, and comprehensive documentation platforms for mental health indicators), as well as barriers on the staff level like significant workloads. A negative feedback loop between the providers and the clinic emerges when these barriers complicate their ability to meet viral suppression goals for PLHIV with mental distress. The providers navigate this negative feedback loop by leveraging facilitators on the clinic level, like decentralized follow-up services.

#### Institutional and system resource limitations are barriers for providing mental health support.

A clinical officer at Clinic B describes how HIV treatment protocols provide limited technical direction on how to address a patient with a mental health comorbidity. He mentions:

*“There are no protocols developed when you have an HIV patient who has depression or any mental health problem. You just screen [for HIV and HTN]. And then think ‘Can the counselor handle [the patient]?’ If they can’t, then you refer them externally. (PID 9, Clinic B, Clinical Officer)*

The clinical officer’s ability to help his mentally distressed patients is complicated by the limited mental health specific protocols in the HIV clinic (*inner setting, CFIR).* In the absence of these resources, when the clinical officer encounters a patient with mental distress, he mentions relying on the psychosocial counselor in the clinic and her ability to “handle” the patient. The decision to “handle” a patient with mental distress largely relies on how much time the psychosocial provider has that day and the overall behavior of the patient (*decision making, TDF)*. Typically, patients who exhibit *“unruly behavior” (PID 3, Clinic A, Nurse Counselor)* are automatically referred outside of the HIV clinic, to a psychiatric clinical officer or higher-level mental health facility. Days where the provider has limited time due to excess patient flow means patients are typically referred externally as well. External referrals of PLHIV with mental distress means ensuring long term continuity of care is exceedingly difficult, and this influences the providers’ ability to ensure that their PLHIV achieve viral load suppression:

*“It [mental health specific protocols] may also help us clearly identify some issues before they are in the later stages. For example, if someone abuses alcohol [...] It’s hard for them to have a suppressed viral load. So mainly, it would help us. I would know how to help the patient suppress [HIV viral load], and I would reach my viral suppression goal.” (PID 3, Clinic A, Clinical Officer)*

The clinical officer here describes the importance of identifying mental health conditions among her patients upon an early onset. For this provider, addressing these mental health needs is intrinsically tied to delivering HIV specific care. Early identification and management of mental distress, like alcohol misuse, will serve as facilitators to better counsel patients on how they can achieve viral suppression, a professional responsibility she has (*goal/target setting, TDF)*. Achieving her professional goals, then, is dependent upon enhancing the resource limitations within her own context (*knowledge, TDF*) and the resource limitations within the clinic’s context.

All the providers expressed that HIV implementing programs must incorporate mental health specific protocols, guidelines, and expertise in their support to help providers facilitate positive mental health outcomes for PLHIV. This structural presence, then, has implications for how providers can ensure their patients achieve viral suppression. While experiencing ambiguity and helplessness in the lack of these resources, though, they adapt and “make it work” to reach these targets.

#### Providers’ motivation to achieve optimal viral load suppression to overcome barriers for providing mental health support.

Despite experiencing a negative feedback loop with the clinic, providers achieve their professional goals of viral load suppression by giving their own resources to support the inner setting features they need. These efforts to “make it work,” however, still demand additional infrastructural support to achieve their fullest potential. For example, a nurse counselor at Clinic B describes an encounter she had with a visibly distressed patient on an active clinic day with a higher workload:

*[R]: “We didn’t have a space for her. We didn’t have a person inside [no mental health focal person or specialist] for her. Instead, we gave her drugs to sedate her. We tried to lock her in a small room. Then we have a psychiatric clinical officer here. So, after we referred her to that person outside of the clinic.*

*[I]: “So after you referred her to the psychiatric officer, were you able to follow up with her?”*

*[R]: “Sometimes it’s marked on the document [patient HIV monitoring card]. Sometimes I find a relative and ask them how they are. But they say [the patient] is improving. [...] Sometimes we cannot follow up for adherence and it is difficult to see them again.” (PID 16, Clinic B, Nurse Counselor)*

The nurse documents great difficulties in trying to meet her professional duty of ensuring the mentally distressed patient achieves viral suppression. Where inner setting facilitators like in-clinic expertise, relevant medications, protocols, and necessary space were missing, the nurse unwillingly reconciled with the existing infrastructure of the clinic herself. The nurse counselor offered sedatives and found a separate room to adequately manage the patient internally. In these circumstances, she continues to “make it work” by contacting relatives and performing follow up services. She performs patient follow up herself to ensure her patient is adhering to their ARV medications. These attempts are often futile, complicating her ability to achieve these professional goals.

By contributing these inner setting resources from her own context, the provider notes feeling “overloaded,” and her professional boundaries are stretched thin (*Professional Boundary, TDF)*. Another nurse counselor similarly describes the added efforts she undertakes to cater to a patient’s emotional needs:

*“Others [patients] that don’t come to the hospital, I must reach out to them at home or wherever they may be. So that they feel catered for and not alone in these things. Because of this, the workload increases. They [hospital administration] measure the performance of the department. They see these people [PLHIV] are not taking the medications well. So performance… it affects us.” (PID 6, Clinic B, Nurse Counsellor).*

At the expense of her own personal time and limited organizational resources, the nurse counselor engages in community level follow up to ensure patients are adhering to medications. She describes how the management of the hospital measures the performance of the clinic based on how many patients are virally suppressed. The limited inner setting features available to address the mental health burden among the patients means bridging the gaps come from resources in her own context, like using her own time and money to perform community outreach.

All the participants expressed a commitment to navigating within this HIV clinic/provider negative feedback loop to achieve their professional goals and cater to patients’ emotional needs. This meant providers supplement existing inner and outer setting resources with ones from their own contexts. Optimizing vertical features of HIV programming with clinic-specific mental health protocols, mental health focal persons, and relevant medications can disrupt this negative feedback loop and better support the providers commitment to “making it work.”

### Providers optimize ARV adherence counseling to respond to PLHIV’s mental distress

A key facilitator to the integration of mental healthcare service delivery in the provider context is that providers have vast interpretations of mental health problems among their patient populations. Because of this, they are perceptive to their patients’ comport and can identify when additional psychosocial support is needed. For example, some summarized their mentally distressed patients as those who exhibit behaviors like *“disorganized speech, excessive talking, appearing confused, or dirty, or crying” (PID 10, Clinic B, Nurse Counsellor).* Others mentioned encountering patients with more invisible mental distress, like *“suicide, sad, or stress” (PID 2, Clinic A, Nurse Counsellor),* which is not readily noticeable to the provider, unless indicated by the patient.

A key barrier in the clinic context, however, is the limited scope for psychosocial assessments within adherence counseling. A universal responsibility among the providers, adherence counseling is understanding *“what the people are going through” (PID 1, Clinic A, Psychosocial Counsellor)* that impedes their ability to adhere to their antiretroviral (ARV) medications. Typically, these interactions are understanding what behaviors are impacting the patient’s ability to adhere to ARVs and how they can be modified to achieve viral suppression. A negative feedback loop between the provider and the patients they treat emerges when the providers are perceptive to their patients’ mental distress, but do not have the adequate tools in the clinic to address it. Providers navigate this negative feedback loop by leaning on religion, empathy, and visualizing innovative solutions to “make it work” with adherence counseling.

#### Adherence counseling is insufficient in addressing complex mental distress.

A clinical officer at Clinic B describes an encounter with a patient struggling with ARV medication adherence. She started engaging the patient in adherence counseling sessions to understand why the patient was not adhering to treatment. This consisted of explaining the importance of taking ARVs on time and the negative health impacts of failing to do so. These interactions largely consisted of the patient placating the provider, saying that he will adhere moving forward. Subsequent interactions, however, revealed that he was still struggling with adherence. The provider eventually uncovered deeper mental distress.

*“He said a woman raped him. So, he was very bitter. He became very depressed. So, he took the whole thing of ARVs [swallowed a whole monthly supply of ARVs]. He was brought here [to the HIV clinic] for support, but he passed [died] on the third day. That is bad. Depression can do a lot of different things. It is a little hard to see it [mental distress] sometimes.” (PID 7, Clinic B, Clinical Officer)*

This patient fit the clinical officer’s perceptions of mental distress, in that he appeared bitter and was very quiet *(Schemas, TDF)*. What was not readily apparent through adherence counseling interactions, however, was the patient’s suicidality and his intent to act on his suicidal ideation. Deeper intervention outside of the clinical setting provided the clinic officer more context for the patient’s mental health *(Outer Setting, CFIR),* but ultimately, she required more than adherence counseling to address these issues in the clinic. While a facilitator in this interaction was the provider’s ability to recognize that the patient was likely experiencing complex mental distress, she ultimately was not able to uncover his invisible suicidality until he passed away.

It is important to note, however, that providers regularly encounter patients who are open about the mental distress that they are facing, making it easier for them to identify and adequately acknowledge. In many of these cases though, adherence counseling is still insufficient in catering to the explicit mental health needs patients express. A provider describes a time when she saw a patient who was mentally distressed after testing positive for HIV. The patient was to be married off soon, and the positive test had meant that the wedding would not happen.

*“She was very depressed […] So she cried her heart out and she didn’t know what to do. And we had nothing to do for her. We only took her through [adherence] counseling. We told her she had to tell the man her results, we told her to come back with the man, so we can test him and see [if he had HIV]. So, it was hard for us, we didn’t know how we could help her.” (PID 6, Clinic B, Nurse Counsellor)*

In this case, the patient was verbal about her distress and readily expressed the deeper contextual problems (*Outer Setting, CFIR)* that contributed to it. The nurse counselor described how linking the patient to HIV care and engaging in adherence counseling was not enough to assuage the patient and relieve her of her emotional turmoil. Despite fully knowing and understanding the extent to which the woman was experiencing mental distress, the provider did not feel adequately resourced to address it (*Self Efficacy, TDF)*.

The consequences of adherence counseling not meeting complex emotional needs leads to patients passing away and the provider losing ability to contact and reach them, despite the fact providers are perceptive to their mental distress. To navigate this negative feedback loop with the patients, they rely on religion, empathy, and generate innovative solutions to improve the adherence counseling process.

#### Providers employ inventive problem-solving to enhance adherence counseling.

There are a host of facilitators in the provider context, that if leveraged appropriately, can overcome the limitations of adherence counseling. For example, a provider at Clinic B describes an interaction with a patient who tested positive for HIV. The patient was upset that he had contracted the virus from his wife and felt that she had been unfaithful to him. The patient was successfully linked to care and started taking medications accordingly. After multiple adherence counseling interactions, the patient willingly revealed to the provider that he “wanted to kill himself.” The provider responded:

*“I just talked about God. I’m like ‘forgiveness is good. Do you go to church? Now killing yourself is not an option.’ I just became some lady who listened to him. Somehow, we spent around 2 hours talking. By the time he left here [the clinic], he was happy. I felt happy…. he asked for my number and started calling me. He calls me and says, ‘Omusawo, you helped me a lot.’ I showed him another side of life.” (PID 13, Clinic B, Clinical Officer)*

The clinical officer indicated that she did not have the clinical expertise to guide the patient through his depression and subsequent suicidal ideation *(Skills, TDF).* She recognized a need, however, to address the patient’s social context beyond medication adherence. She relied on religious teachings to instill hope in the patient. She stepped out of her role as a healthcare provider and became someone the patient can rely on within and outside of the clinic as well (*Professional Roles/Boundaries, TDF)*. These hidden, additional efforts outside of her traditional responsibilities facilitated positive mental health outcomes for the patient. For some providers, however, “making it work” with adherence counseling was not a process that emerged out of not knowing what else can be done. Many times, they readily had innovative ideas to improve mental health outcomes, and felt confident in their ability to do so within this context:

*“I can help them [mentally distressed patients]. Some of them have gardens, so I let them know that they can grow things, and then they can start earning money that way. Some of them are depressed over money issues. So, if they don’t have somewhere to get the money, they just tend to drink cheap alcohol to pass time. So, I try providing them other innovations.” (PID 10, Clinic B, Psychosocial Counselor)*

To address the deeper social context that contributes to a patient’s depression or alcohol use disorder, the provider relies on understanding other aspects of the patient’s life that can counteract and facilitate healthier outcomes *(Outer Setting, CFIR)*. If she notices that a patient has a garden, she recommends trying to garden and farm vegetables as a means for the patient to find purpose and earnings to feel empowered. The provider indicated that though she can confidently make these recommendations, she requires additional support on guiding PLHIV to practicing them.

Overall, HIV care providers encounter patients experiencing a wide spectrum of mental distress. In these circumstances, adherence counseling is often insufficient in addressing patient mental health needs. Providers “make it work” with varying levels of confidence. This includes relying on religious teachings, striving to meet their patients with empathy, and proposing innovative solutions. In doing so, they step outside of their traditional responsibilities as healthcare providers and experience added stress.

### Unrealistic organizational and cultural demands are barriers to mental health service provision

The providers noted that while navigating the negative feedback loops with the clinics and the patients, they recognize unrealistic expectations placed both on themselves. The nurse counselor at Clinic B mentioned an unrealistic demand placed upon her when she encounters patients experiencing mental distress:

*“They [PLHIV] feel bad because they thought that we were going to provide food and transport. They thought we could cure HIV. They thought we could do these things… Even ‘fix bewitchment.’ [...] We cannot do those things. But they feel bad. We just counsel them, and they get caught up with the situation.” (PID 12, Clinic B, Nurse Counsellor)*

The provider describes several sociocultural factors that shape a patient’s predisposition to mental distress that she is tasked with addressing. Oftentimes, patients request additional support for food and transport, have difficulties accepting the chronicity of their HIV status, and want providers to address their state of “bewitchment.” (*Outer Setting, CFIR).* These perceptions of bewitchment are common constructions of psychological distress in central Uganda and often lead to impacted individuals feeling ostracized. The nurse counselor notes how in these adherence counseling interactions, she can provide health education and explain the impacts of not adhering, and the patients reconcile with that. Along with having to address “bewitchment,” the psychiatric clinical officer at Clinic A discusses how he reckons with this label as well:

*“Most of the people that come to see us are stigmatized. Sometimes, they remain in the community. They [patients with mental health problems] fear coming to us. Community calls them omulalu, and sometimes they call us, the psychiatric providers, omulalu. Even if you try to see them in their home, they will not open their doors for you.” (PID 4, Psychiatric Clinical Officer, Clinic A*).”

Along with being bewitched, patients with mental health problems are construed and labeled as *omulalu*. It is a Luganda word encompassing observed behaviors that are considered or perceived to be socially abnormal. The term encompasses simple outbursts to more severe manifestations of mental illness. It holds a deeper significance as a stigmatizing word relating to *madness* (*Outer Setting, CFIR).* Fearing this socially isolating label, many patients suffering from distress do not openly seek care and resist coming to the clinic for support. This places the onus on the provider to actively seek these patients in the communities, which is difficult to achieve because the providers are not readily received for help and support. The psychiatric clinic officer also indicated that in providing mental health related support to patients, he himself has been termed as *omulalu*. He accepts such labels as “normal,” but acknowledges that he faces an unrealistic demand to resist these long-standing communal stigmas to provide mental healthcare.

Overall, providers indicated that operating within the fullest extent of their context and going above and beyond is sometimes not enough to facilitate positive mental health or holistic health outcomes. The unrealistic expectations on themselves and their patients imposes significant barriers to both provide mental healthcare and receive it. Sometimes, *“you [the provider] can educate them, do all you can, and at the end they [the patient] will say ‘I will take my herbal medicines.’”* (PID 3, Clinic A, Clinical Officer). If the patients feel that their needs are not met in the clinic, they seek alternative sources of care, and this can negatively impact their mental health problems and abilities adherence to ARV medications. Patients are often seeking non biomedical solutions to their circumstances, hindering a provider’s ability to provide adequate care..

The providers documented a variety of procedures that ease the complex sociocultural and organizational burdens placed upon them. The clinical officer at the public facility describes a stark change she noticed among her patients’ ability to open up to her after a medical doctor with HIV and HTN, who does not work at the health center, came to speak to the patients about his journey with these conditions. She described:

*“The doctor told the patients, ‘If you have any problems, tell the clinical officer.’ When he expressed this to them, the patients were happy. When we received them, they opened up more, they said they will come back next time for more treatment. It’s important for them to have someone who understands their problems tell them that ‘it will be okay’.” (PID 3, Clinic A, Clinical Officer)*

The clinical officer recognizes that a limitation she cannot overcome is shared experience with the patients who have a disease. After a reputed individual with HIV and HTN spoke to the patients about how they can live with their chronic conditions and how the healthcare providers can support them, this burden was lifted from the provider, and she could better tend to her patients’ emotional needs. The providers generally recognize that there are some patient needs they cannot meet through “making it work” without formalized integrated mental healthcare services. They are frequently tasked with addressing larger, systemic issues like poverty and cultural stigmas. The providers discuss how certain processes like relying on expert clients and facilitating social support mechanisms overcome these demands, but they feel uncomfortable to go the certain lengths they do to leverage these mechanisms.

### Providers *lose it* as a consequence of “making it work.”

HIV healthcare providers, in “making it work” and while experiencing unrealistic demands in doing so, describe themselves as *“basically depressed and traumatized”* (*PID 6, Clinic B, Nurse Counsellor)*. They discuss how oftentimes; they know and understand the appropriate course of action to take when they encounter patients experiencing mental distress but feel constrained in their ability to do so. For instance, a nurse counselor at Clinic B indicates:

*“Someone can say to you: ‘leave me to die.’ That person will not listen to anything. ‘Leave me to die.’ If you hear those statements, you know that you must put more effort there. That is just one sentence, but it means a lot. In your heart, you don’t feel good. You know there is not much you can do for them.” (PID 10, Clinic B, Nurse Counsellor)*

The provider describes feeling a sense of dread whenever she hears patients wanting to be left to die *(Anticipated Regret, TDF)*. The nurse counselor understands the impact an HIV diagnosis has on the patient’s own psyche and reflects on the fact that she is limited in her capability to address the mental distress. Patients showing signs of suicidal ideation require many additional efforts to probe into their mental health, and as previously mentioned, these additional efforts are beyond their cadre, training, ability. Providers additionally documented these experiences long after their interactions with their patients as well.

*“There was this lady, she came to test for HIV. She was raped by her boss. And then she was told to not tell anyone. Then she was like Omusawo [healthcare provider] ‘why is it that that guy raped me?’ That lady was like losing it. We couldn’t manage her because she moved away. We can’t afford to look for her. I lose it when I think about her.” (PID 14, Clinic B, Laboratory Technician)*

The provider describes living with the guilt that she could not do more for her patients considering the constraints that she is working within. She describes internalizing the psychosocial pain that the patient presents. Within the context of limited resources, the provider knows that there is not much she can do for the patient and lives with that guilt every day.

As providers cycle through these negative feedback loops, they experience the cumulative deprivation of resource limitations, feel constrained in their abilities to comprehensively tend to patient needs, and internalize their patients’ pain as well. This results in the depletion of their own context. So much so, that they want to “*leave healthcare, and go and do other things.” (PID 6, Clinic B, Nurse Counselor)*. Providers frequently *“go home exhausted, come back [to the clinic] exhausted, and leave again exhausted” (PID 1, Clinic A, Psychosocial Counselor)*. In the absence of formalized mental health services, the providers find themselves in perpetual, cyclical, context-depleting cycles that impact their own quality of life.

## Discussion

This study sought to explore how HIV providers address the mental health needs of their patients in the context of integrated HTN and HIV services. While this study is not focused on integrated hypertension care, provider reflections on their experience with HTN/HIV integration enriched their understanding of what may be required for successful mental health integration and served as an important reference point in our analysis. Through this exploration, we identified that formalized mental health services (i.e., mental health specific protocols, screening tools, and expertise) are not present at either of the two HIV clinics. There are, however, other infrastructural resources available at the clinics that providers leverage to address PLHIV’s mental health needs. These include peer counselors, who perform decentralized follow up services, as well as nurse counselors, who have dedicated psychosocial training meant to enable ARV adherence. Providers anticipate their patients’ mental health needs and acknowledge emerging tendencies of suicidality, depression, and alcohol abuse among PLHIV. Notably, they establish and perceive that mental health problems arise only among those who struggle with viral suppression, but future integration efforts should consider patients who are successfully adhering to ARVs. Given their perceptions that treating mental health issues is vital for achieving HIV viral suppression targets, they leverage the aforementioned HIV care cascade characteristics, among others, to address PLHIV’s mental health needs. In the absence of formalized integrated mental health services, however, providers experience tensions with the HIV clinics they work for and the patients they serve. These tensions can be conceptualized as negative feedback loops, with the providers situated in the middle. They describe “making it work” within these negative feedback loops to cater to their patients’ mental health needs, at the expense of their own personal boundaries with their work and larger sociocultural limitations as well.

### Facilitating a positive clinic and provider feedback loop

Our findings revealed that HIV providers experience a negative feedback loop with the HIV clinic because of the few infrastructural resources at the clinic level available to cater to PLHIV’s mental health needs. These include limited mental service delivery protocols, platforms where mental health indicators are tracked and monitored along the care continuum, and especially in public facilities, limited private spaces to ask sensitive mental health related questions. These barriers culminate and make it difficult for providers to ensure their PLHIV with mental distress achieve viral suppression, a key performance metric for the provider and the clinics at large. Providers adapt within this context by performing patient outreach services outside of the health facility on their own time, money, and resources to achieve their professional responsibilities. Facilitating a positive clinic and provider feedback loop, one which provides adequate clinic level resources to address mental health needs, is vital to successfully integrating mental healthcare. In terms of enhancing clinic specific guidelines or protocols on mental health services integration, prior research in Uganda suggests that a protocolized clinical depression screening model, in which validated tools are used for assessing mental health, yielded more accurate assessments of depression than relying on subjective clinical acumen [20]. Integrating these assessments within HIV/HTN and other care cascades can provide technical support in addressing patient mental health needs and holistically support PLHIV outcomes, without straining provider resources.

### Facilitating a positive patient and provider feedback loop

Providers are perceptive to PLHIV’s mental distress; however, they feel ill-equipped to address this distress in the clinical setting. The most salient feature in this setting to address mental distress is adherence counseling, a practice by which nurse counselors work one-on-one with patients to understand what challenges patients face in adhering to ARVs. Provider narratives indicate that adherence counseling is sometimes insufficient in appraising deep emotional distress like suicidality. Sometimes, providers who have the capacity to, overcome the limitations of adherence counseling with various inventive problem-solving strategies, that if protocolized, can aid them in their diagnostic efforts. Previously in Uganda, a savings-led family based economic empowerment intervention has successfully improved adolescents linkage and adherence to ART [21]. Our findings corroborate that financial insecurity is a determinant of mental distress, and providers address it by augmenting adherence counseling with economic empowerment recommendations like home gardening. Protocolizing these practices that providers already engage in can support mental health integration efforts. Additionally, prior literature suggests that culturally relevant psychosocial programming for PLHIV, especially ones with racially congruent images and framing, are more effective in improving mental health outcomes [22]. Including religiosity in such culturally relevant programming can especially aid both PLHIV and their providers in improving mental health outcomes.

Additionally, interdisciplinary efforts have shown promising success in improving PLHIV mental health needs. A screening integration effort in Zimbabwe capitalizing on nurses, community health workers, traditional medicine practitioners, and routine HIV service providers in clinics reported a more balanced work load among the care team. They also reported more streamlined uptake of referral pathways and improved relationships with their patients [23]. This further highlights our findings that when there is cohesion among clinical systems and the providers, relationships with patients improve and mental health needs can be more adequately cared for.

### Overcoming the insurmountable conditions of the clinic-provider-patient negative feedback loops

While navigating these negative feedback loops with the clinic and the patients, the providers note unrealistic demands placed upon both them and their patients. First, they describe having to contend with longstanding communal and interpersonal mental health stigmas. They note that culturally constructed mental health stigmas prevent patients from seeking mental health specific care, and that many stigmatize those who provide formalized mental health services as well. Stigma reduction approaches must be considered in integrated HIV and mental health care. The providers have relied on peer clients to appeal to patients to talk about their chronic conditions openly and honestly with them, and this has showcased some success. In Botswana, a stigma reduction approach through leveraging peer counselors to establish rapport reduced the burden on both the healthcare provider and the HIV positive patient [24]. Additionally, other peer interventions have shown high acceptability among young mothers with HIV in Uganda, especially with mental health related issues. Our findings demonstrate that peers or expert clients are critical in linking PLHIV to integrated HTN care, and these facilitators can be leveraged for mental healthcare as well.

Adapting within these negative feedback loops poses a significant toll on the providers’ own mental health. They describe feelings of longstanding, residual guilt after being unable to provide comprehensive care to their patients. A lack of formalized mental health services has resulted in patients losing contact with their providers and in some cases, passing away. Prior ethnographic research in Uganda suggests that nurses and other healthcare providers, in the absence of adequate resources to provide comprehensive care to patients, experience moral distress. Despite knowing and wanting to take ethically appropriate actions, the providers are limited in their ability to actually do so [25]. This is particularly true in the HIV context, as nurses and other providers pull from their personal resources to cater to complex patient needs, and vicariously experience the long-term impacts of not meeting them.

Implementation strategies for integrated mental health and HIV services can leverage the providers’ innate desires to want to help their patients overcome these needs and institute the appropriate supervision systems and mental health support to achieve these goals. Specifically, strategies such as provider training in mental health screening and communication, ongoing supervision and mentorship from specialists, routine feedback loops, and integrating screening tools into HIV care workflows can help institutionalize what providers are already engaging in formally. These strategies build on providers’ existing commitment and resourcefulness, and help scale sustainable, low resource approaches to delivering mental health support without requiring entirely new interventions. When incorporated within clinical settings and existing integrated models of care, these strategies can create sustainable environments for providers to deliver comprehensive mental healthcare.

## Conclusion

Despite the fact that HIV treatment centers in Uganda are integrating chronic disease care, mental health services are largely missing from these clinics. This is especially critical, given the high burden of mental health issues like suicidality, alcohol use, and depression among PLHIV. Providers note that in the absence of mental healthcare in HIV clinics, they toil within resource limitations to address their patients complex mental health needs. Overcoming these infrastructural and interpersonal resource limitations places a strain on the providers. The results highlight the need for context specific implementation strategies that leverage the invisible work the providers already engage with in a sustainable manner.

## Limitations

This study has two limitations worth noting. The interviews at the public facility were oftentimes done in open spaces, in the presence of other providers and patients due to the lack of privacy in the facility. This may have impacted how participants responded to the questions and the comfort with which they were able to be transparent. We tried to overcome this limitation by conducting some of the interviews outside of the clinic, but within the premises of the health facility on days where there was minimal foot traffic. Additionally, the research team believes that thematic, code, and meaning saturation has been achieved, through iterative coding and debriefing sessions. The clinics, in terms of services provided and staffing, were largely representative of other public and private funded facilities providing HIV and comprehensive NCD care. It is important to note that a large majority of the participants were female and nurse counselors. While most clinic settings have this makeup, male respondents might have different perspectives that were not captured in this dataset. This may impact the generalizability of the findings to providers in other clinical settings in Uganda.

## Supporting information

S1 File Interview Guide. Interview guide used during semi-structured interviews.
(DOCX)

## Abbreviations

ART: Antiretroviral Therapy
ARV: Antiretroviral (medication)
CFIR: Consolidated Framework for Implementation Research
HIPAA: Health Insurance Portability and Accountability Act
HTN: Hypertension
HTN/HIV: Hypertension and HIV (integrated care context)
HIV: Human Immunodeficiency Virus
IRB: Institutional Review Board
Mak SOMREC: Makerere University School of Medicine and Research Ethics Committee
NCD: Noncommunicable Disease
PEPFAR: US President’s Emergency Plan for AIDS Relief
PID: Participant Identifier
PLHIV: People living with HIV
PULESA: Strengthening the Blood Pressure Care and Treatment Cascade for Ugandans Living with HIV– Implementation Strategies to Save Lives
TDF: Theoretical Domains Framework
UNCST: Uganda National Council of Science and Technology
