# Supplementary material for: “Just trying to make it work:” A qualitative exploration of healthcare provider implementation considerations for integrating mental health and HIV care in central Uganda
Source: PLoS One. 2026 Aug 14;21(8):e0353859. doi: 10.1371/journal.pone.0353859 (PMC13475888; doi:10.1371/journal.pone.0353859)
Supplement: Supplementary file 1 [file pone.0353859.s001.docx]

### *Semi Structured Interview Guide*

**Domain 1: Background and Context**

**Purpose: Understand the provider’s background, role in providing healthcare, and relationship to the health center.**

1) Tell me a little about what role you play in the hospital.
   a. Probe: What are your responsibilities in delivering patient care for patients with unsuppressed viral loads?
   b. Probe: What are your responsibilities in delivering patient care for patients with suppressed viral loads?
2) What would you consider the hardest part of your job, and why?
   a) Probe: How do you push yourself through the hardest part of your job? What motivates you?
   b) Probe: What is some of the information you have to document? How does it impact you and your patients?
3) So you work alongside nurses, peer educators, counselors, and linkage facilitators. Can you describe how you work with each of these individuals to provide patient care?
   a) Probe: For suppressed patients?
   b) Probe: For unsuppressed patients?

**Domain 2: Provider Conceptualization of and Orientation to Mental Health**

**Purpose: Explore how the provider defines and understands mental health, and what informs this understanding**

4) When was the last time you referred someone to the clinic officer, external mental health services, psychosocial counselor, or psychiatric clinic officer because a patient had a mental health issue?
   a) Why did you refer them?
   b) How often do you do this?
5) Can you describe a time when you received mental health based training? What was talked about and how was it helpful or unhelpful?

**Domain 3: Provider Confidence in Appraising Mental Health Problems**

**Purpose: Understand how confident and prepared the provider feels to address mental health burdens among PLHIV**

6) When was the last time a patient asked you to do something, or care for something that you were not able to?
   a) Probe: What are some of the difficulties in meeting these expectations?
   b) Probe: How about a time when a patient expressed a concern that you were able to meet well?
7) Have you ever seen a patient who was depressed or [insert words participant used to describe mental health]? What kind of conversations did you have with the patient, and what were their reactions?
   a) Probe: In what context did the patient mention this difficulty (i.e. social stigma, treatment adherence, etc.)
   b) Probe: What were you thinking about when you were trying to counsel or address the patient?
   c) Probe: How did the patient respond to the conversation and your counseling?
   d) Probe: What happened to the patient after the interaction?
8) Have you ever talked to family members of patients who struggle with medication adherence or any mental health problems? What do these conversations look like?
   a) Probe: What are some of the challenges associated with talking to family members?
9) Can you tell me about a time you felt supported by your team in delivering care?
   a) Probe: How about a time you felt unsupported?

**Domain 4: Evaluating Existing Protocols and Procedures for Mental Health Support**

**Purpose: Understand what factors might influence the adoption of, adherence to, and maintenance of mental health service provision**

10) How do you screen for mental health problems among patients with HIV/AIDS?
    a) Probe: How do you know when someone has a mental illness?
11) What protocols or referral processes are in place for patients who express or display signs of mental distress?
    a) Probe: What are internal referral processes within the ART clinic and hospital?
    b) Probe: What are the external referral processes to care sources outside of the hospital?
12) What existing tools, documentation platforms, or clinical processes are helpful in tracking and monitoring HIV/HTN care, that you think can be applied for tracking and monitoring patient mental health as well?
    a) Probe: What makes these tools, documentation platforms, and clinical processes helpful?
13) What are some of the capacity or organizational constraints that would limit the extent to which mental health screening, support, and care services are provided?

**Domain 5: Provider Perceptions of Integrated Mental Health with HIV/HTN Care**

**Purpose: Evaluate the perceived need and impact integrated mental health with HIV/HTN care would have on clinical burdens, and whether there is adequate administrative support to carry out such integration**

14) Which aspects of providing care do you prioritize during the time you spend with your patients, and why?
15) When the process of integrating HIV and HTN care started, what tensions did you notice and what helped the team overcome them?
    a) Probe: What are some of the advantages of integrated HIV/HTN care, for you, specifically?
    b) Probe: What are some of the disadvantages of integrated HIV/HTN care, for you, specifically?
16) Within the current constraints of the HIV/HTN care cascade, what scope is there to screen patients for a mental health morbidity?
    a) Probe: Who should be responsible for screening, and why?
    b) Probe: What type of administrative or infrastructural support would be required from the ART clinic and hospital to perform screening services?
17) What impact would integrated mental health with HIV/HTN care have on your provision of clinical services? (i.e. make things easier, more complicated, etc.)
18) What perceived impact would integrated mental health with HIV/HTN care have on your patients? (i.e. would it improve ART retention, etc.)

**Domain 6: Demographic Questions**

19) What is your age?
20) What is your educational status?
21) What is your job title?
22) How long have you been a healthcare provider?
23) How long have you been working at the health facility?
24) How many patients do you typically offer counseling services to each shift?
25) How much time do you typically spend with each patient when you are counseling them?
